# A Rare Case of Biliary Atresia with Biliary Ascites on a (Hepatobiliary Iminodiacetic Acid) HIDA Scan

**DOI:** 10.1055/s-0043-1764305

**Published:** 2024-04-22

**Authors:** Poonamjeet Kaur Loyal, Khalid Makhdomi, Samuel Gitau

**Affiliations:** 1 Department of Radiology, Aga Khan University Hospital, Nairobi, Kenya; 2 Nuclear Medicine Division, Department of Radiology, Aga Khan University Hospital, Nairobi, Kenya; 3 PET/CT Imaging, Department of Radiology, Aga Khan University Hospital, Nairobi, Kenya

**Keywords:** Biliary atresia, biliary ascites, HIDA

## Abstract

Biliary atresia is one of the most challenging conditions in pediatric surgery even when it is the only finding. Here we present a rare case of biliary atresia complicated with biliary ascites due to ductal perforation identified on a hepatobiliary iminodiacetic acid (HIDA) scan.

## Introduction

Biliary atresia is the most common neonatal cholestatic disorder with a varying incidence of 1:8000 to 1:18000 and a slight female preponderance of 1.4:1 affecting all races. 1 Most patients, up to 75 to 90%, have no other congenital anomaly and are classified as “classical biliary atresia,” whereas the remaining may have a myriad of congenital anomalies including polysplenia, abdominal situs inversus, intrapulmonary shunting, asplenia, pancreatic anomalies, absence of inferior vena cava, and congenital heart disease, portal vein abnormalities, and intestinal malrotation. 2 3 We present a case of biliary atresia compounded by biliary ascites.

## Case Report

A 1-month-old child had progressive abdominal distention since birth associated with pale stools, dark urine, tense ascites and scleral jaundice. The patient also had an element of peritonitis on the clinical exam. Abdominal ultrasound showed tense ascites and the gall bladder was not visualized. The patient was referred to our facility for a HIDA scan.

In all, 33 MBq of ^99m^ Tc-DISIDA was injected IV and dynamic images of the abdomen acquired for 1 hour. Delayed static and single-photon emission computerized tomography/computerized tomography (SPECT/CT) images of the abdomen were acquired at 2 hours.

**Fig. 1:**
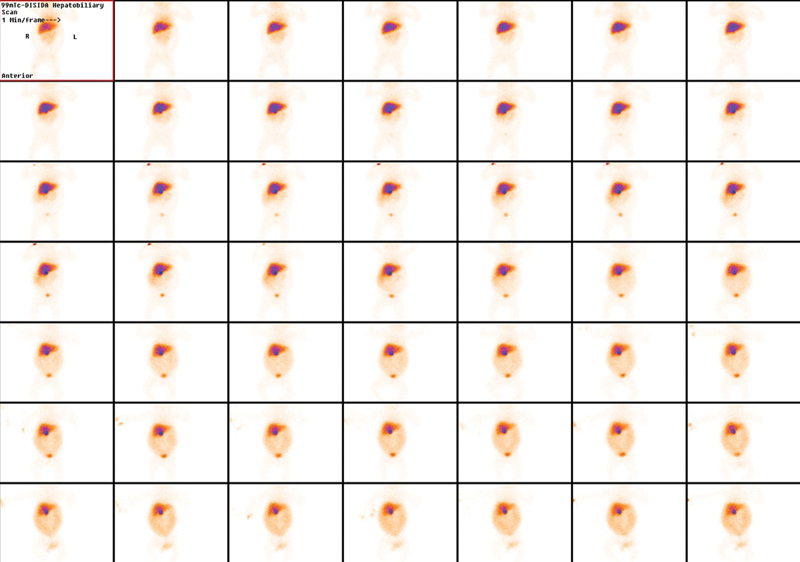
Dynamic flow images (15 frames at 4 seconds/frame, 64 × 64 matrix) of the abdomen demonstrating gradual diffuse accumulation in the peritoneal cavity.

**Fig. 2:**
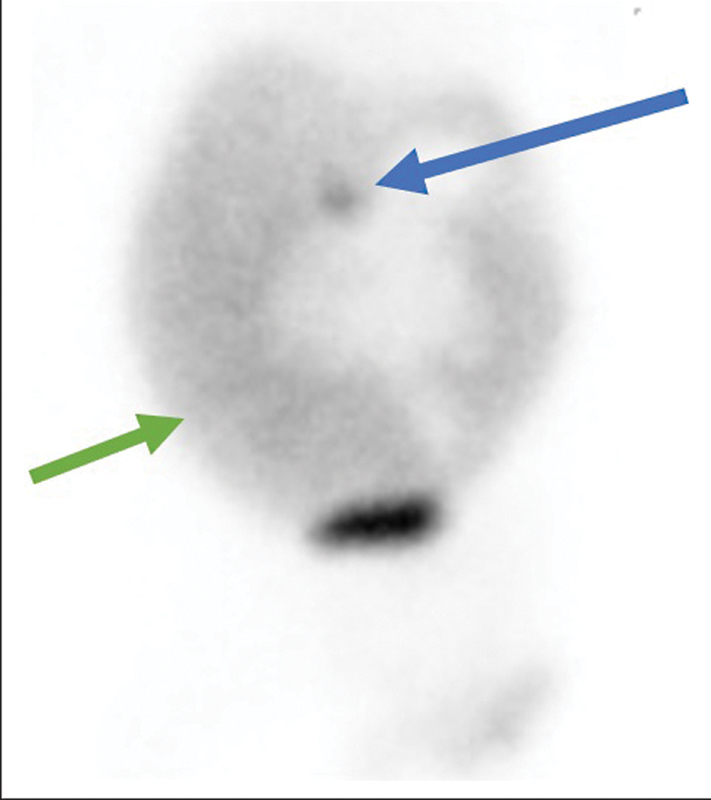
Delayed planar image at 6 hours showing focal tracer accumulation in the liver ( *blue arrow* ) and tracer accumulation in the peritoneal activity ( *green* ).

There was normal and fairly uniform hepatic parenchymal tracer uptake. Tracer excretion into the intestines was not visualized even on the delayed images, which was concerning for biliary atresia. There was, however, progressive tracer accumulation in the ascitic fluid ( Fig. 1 ). A focus of tracer stasis was noted from the 10th minute of the dynamic study at the inferior margin of the right lobe of the liver, which intraoperatively was confirmed to be the site of the leak ( Figs. 2 3 4 ).

**Fig. 3:**
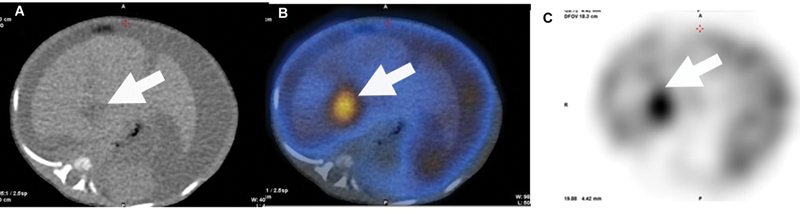
Low-dose CT ( **A** ) corresponding fused SPECT/CT ( **B** ) and SPECT images ( **C** ). In Fig. 2A , there is a hypodensity in the liver that demonstrates tracer pooling on the SPECT/CT image **B** and SPECT image **C** . *White arrow* demonstrates the site of possible leak.

**Fig. 4:**
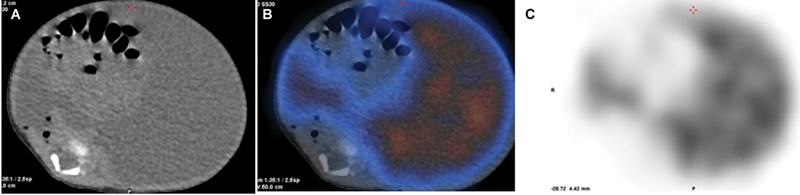
Low-dose CT ( **A** ) corresponding fused SPECT/CT ( **B** ) and SPECT images ( **C** ). These demonstrate the ascites with tracer accumulation.

These findings were confirmed intraoperatively, which was done at an external facility. The patient is currently doing well and has recovered fully.

## Discussion

Biliary atresia is an inflammatory fibro-oliterative cholangiopathy associated with progressive fibrosis affecting the intrahepatic and extrahepatic biliary ducts. This eventually leads to portal hypertension, liver failure, and invariably death in the first 2 years. 4 The pathophysiology remains unknown but has been attributed to intrauterine and perinatal injury to the bile ducts, which may be due to vascular injury, infections, e.g., viruses, genetic predisposition, and several environmental factors including toxins, alcohol, and amphetamines, followed by immune-related injury. 5 6 There are two interventions available for treatment including a portoenterostomy, also known as the Kasai procedure named after the Japanese surgeon Moroi Kasai who first performed it in 1950 and the second was liver transplantation. 7 8

Although there are many reports in the literature that describe biliary leak post intervention, but the development of the same before surgery is less frequently seen. Similar to our case, there have been reports in the literature of hepatic ductal perforation associated with biliary ascites and jaundice. 9 It is postulated that in the perinatal period, the biliary flow increases and hence this leads to tissue damage. This eventually leads to bile leakage, impaired biliary flow, and further trigger inflammatory response and tissue damage. 10 We postulate that this may have been the case in outpatient. HIDA scan can be very useful in characterizing ascites as biliary as was in this case.

This case emphasizes the importance of early diagnosis that guides the intervention resulting in good patient outcomes.

In conclusion, biliary atresia, which is the commonest neonatal cholestatic disorder, is a difficult condition to manage. HIDA scan is a valuable investigation in the work up of patients suspected to have biliary atresia. It is important to be aware of the rare complication of biliary perforation, leading to biliary ascites that may be identified on a HIDA scan.
